# Seroprevalence of sand fly fever Sicilian virus in blood donors in mainland Portugal

**DOI:** 10.1186/s13071-025-06885-x

**Published:** 2025-07-05

**Authors:** Rafael Rocha, Elif Kurum, Nazli Ayhan, Rémi Charrel, Carla Maia

**Affiliations:** 1 Global Health and Tropical Medicine (GHTM), Associate Laboratory in Translation and Innovation towards Global Health, LA-REAL, Instituto de Higiene e Medicina Tropical (IHMT), Universidade Nova de Lisboa (UNL), Lisbon, Portugal; 2 Unité des Virus Émergents (UVE; Aix-Marseille Univ, Università di Corsica, IRD 190, Inserm 1207, IRBA), Marseille, France; 3 National Reference Center for Arboviruses, Inserm-IRBA, Marseille, France

**Keywords:** Blood donors, *Phlebovirus*, Portugal, Seroprevalence, Sicilian virus

## Abstract

**Background:**

Sicilian virus (SFSV), a phlebovirus transmitted by sand flies, is an understudied arbovirus in the Mediterranean region, with limited data on its epidemiology and human health impact. This study aimed to estimate the seroprevalence of SFSV among blood donors in mainland Portugal and explore associations with sociodemographic factors and exposure to other sand-fly-borne pathogens.

**Methods:**

A cross-sectional study was conducted using serum samples from 800 blood donors collected between February and June 2022. The study sample was selected from a previously established cohort designed for *Leishmania* seroprevalence assessment. The microneutralization technique was employed to detect anti-SFSV antibodies. Sociodemographic data were obtained from self-administered questionnaires. Associations between SFSV seropositivity and Toscana virus (TOSV)/*Leishmania* seropositivity or sociodemographic variables were explored using univariate analysis and multivariate logistic regression.

**Results:**

Overall, the estimated national true seroprevalence of SFSV was 4.7% (95% CI 3.4–6.3%). Regional seroprevalence varied significantly, with the highest rates (up to 11.9%) observed in the Algarve, Alentejo, and Grande Lisboa regions, respectively. In univariate analysis, SFSV seropositivity was not significantly associated with sex, age, dog ownership, or positive serology for TOSV or *Leishmania*. In multivariate analysis, geographic area of residence was the only independent factor associated with seropositivity (adjusted odds ratio 3.05; 95% CI 1.85–5.02; *p* < 0.001).

**Discussion:**

TThis study represents the first nationwide SFSV seroprevalence estimate in Portugal, revealing wider circulation than previously recognized. The lack of association with TOSV or Leishmania seropositivity could suggest the involvement of distinct vector species.

**Conclusions:**

Given the observed geographic clustering, SFSV should be considered in the differential diagnosis of undifferentiated febrile syndromes, particularly in endemic regions during peak sand fly activity. Further research is needed to identify specific vectors, improve diagnostic capabilities, and assess the clinical impact of SFSV infections in Portugal.

**Graphical abstract:**

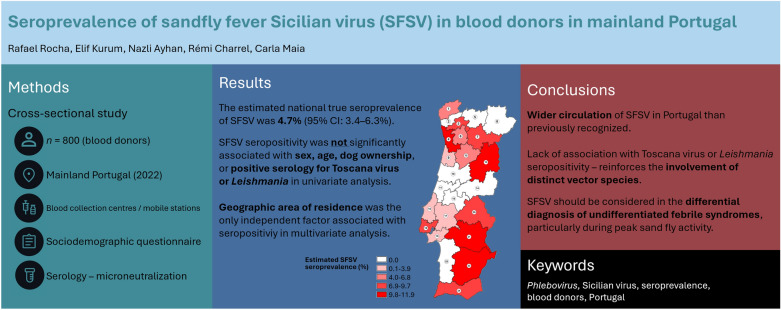

**Supplementary Information:**

The online version contains supplementary material available at 10.1186/s13071-025-06885-x.

## Background

The *Phlebovirus* genus, part of the *Phenuiviridae* family, comprises 68 species [1], some of which are pathogenic to humans. These viruses are transmitted by arthropod vectors, including phlebotomine sand flies [2]. Phlebovirus species transmitted by sand flies may pose an emerging risk to human health, especially as climate change and globalization expand their geographic distribution.

In the Old World, sand-fly-borne phleboviruses with well-documented pathogenic potential in humans are currently classified into three species, all of which co-circulate in the Mediterranean region: Naples virus (SFNV; *Phlebovirus napoliense*), Sicilian virus (SFSV; *Phlebovirus siciliaense*), and Toscana virus (TOSV; *Phlebovirus toscanaense*) [3].

*Phlebotomus papatasi* is the primary vector of SFSV in Mediterranean countries, and transmission occurs through the bite of infected female sand flies, which are most active during the warmer months [4]. Humans are incidental hosts, and the role of wild or domestic mammal reservoirs remains unclear.

In humans, infections with SFSV can present a wide clinical spectrum, though asymptomatic infection appears to be the most common outcome [5]. Among symptomatic cases, the most frequent presentation, known as *sandfly fever* or *Pappataci fever*, typically manifests as a nonspecific, self-limiting febrile syndrome (with an average duration of 3 days), often characterized by headaches, muscle pain, nausea, and vomiting [5].

No studies have specifically assessed the relative contribution of SFSV to undifferentiated fever cases during the warmer months in the Mediterranean. However, serological cross-sectional studies of human and animal populations in confirmed or potentially endemic areas have significantly contributed to the epidemiological understanding of this virus. In the Mediterranean Basin, SFSV seroprevalence studies in humans have shown rates from 0% to 2.2% in Spain [6, 7], from 0% to 1% in southern France and Corsica [8, 9], from 1.1% to 9.2% in Italy [10, 11], around 12.1% in Turkey [12], and around 1.3% in Tunisia [13]. In Portugal, a single seroprevalence study on SFSV, in healthy individuals, reported a value of 4.3% in the Península de Setúbal region [14]. Additionally, only one symptomatic case, in a child, has been reported in Portugal [15]. Finally, in Portugal, recent studies have detected seroprevalence rates ranging from 2.2% to 56.3% for SFSV in dogs and cats across various regions [16–18]. In other countries in the Mediterranean Basin, seroprevalence in domestic animals ranged from 4.5% to 71.9% in dogs (in Kosovo, Greece, and Cyprus) [19, 20] and 0% to 53.4% in livestock (in Kosovo and Egypt) [21, 22]. In wild animals, an SFSV seroprevalence of 45.5% was determined in quails in Spain [23]. However, the experimental infection of dogs with SFSV did not result in disease in these animals, and the detected viremia was low [24]. Thus, dogs do not appear to be natural reservoirs for these viruses nor play a relevant role in the transmission cycle, although they may be exposed to SFSV and serve as sentinels for virus circulation.

Although *P. papatasi* has been identified as SFSV’s vector, SFSV is also detected in *P. ariasi* and the *P. major* complex vector species, which may indicate their contribution to SFSV transmission [25]. While *P. papatasi* has only been sporadically recorded in Portugal, *P. ariasi* is present in several Portuguese regions, including the Algarve, and is detected in higher numbers in higher latitudes (such as the Douro region) and altitudes (such as the Serra da Arrábida). In contrast, *P. perniciosus* appears to be widespread, based on national sand fly surveillance data collected since 2016 [26], and is recognized as the primary vector of *Leishmania infantum* and TOSV in the western Mediterranean [27]. Evidence suggesting co-circulation of SFSV with these pathogens remains scarce.

In this study we aimed to estimate the national seroprevalence of SFSV among blood donors in mainland Portugal through the detection of antibodies using the microneutralization technique. Additionally, the study explored potential associations between anti-SFSV antibodies and the presence of anti-*Leishmania* or anti-TOSV antibodies, as well as various sociodemographic factors, lifestyle practices, and pet ownership statuses among participants.

## Methods

### Study population

Data and samples for this work had previously been collected for another cross-sectional *Leishmania* seroprevalence study [28]. A detailed description of the methodology of the study can be found in Rocha et al. [28], and a summary is provided in Supplementary Table 1. That study targeted the population of people who donate blood in mainland Portugal through the Portuguese Institute of Blood and Transplantation (IPST) or the immunohemotherapy departments (IHDs) of public hospitals in the Alentejo and Algarve regions. Mainland Portugal is located in Southwest Europe, bordering Spain and the Atlantic Ocean, and is divided into 7 Nomenclature of Territorial Units for Statistics (NUTS) 2 regions, 24 NUTS3 regions, and 278 municipalities. According to the 2021 national census, the population of mainland Portugal aged 15–64 years old was 6,257,752 inhabitants [29].

### Data and sample collection

The individuals enrolled in the original study presented to one of the institutions collaborating in the study from February to June 2022 and were considered fit for blood donation. Only individuals aged 18–65 years old were included, reflecting the standard age range established by blood donation protocols. Participant enrollment was performed in non-randomly selected blood collection sessions, but in each session, invitation to participate in the study was random (according to time of presentation at the blood collection center/station). For the original study, each participant filled in a self-administered structured paper questionnaire about sociodemographic aspects; the information collected through this questionnaire was also used in the present study. Additionally, 1.5 mL of the serum routinely collected from each participant was sent to the Instituto de Higiene e Medicina Tropical (IHMT) and stored at –20 °C for the original study. Only participants who consented to be included in further studies were eligible for the SFSV seroprevalence study. Of these (*n* = 3618), a subsample of 800 donors was non-randomly selected, on the basis of the following criteria, to explore potential associations between exposure to different sand-fly-borne pathogens: all the participants who had a positive serology for TOSV (*n* = 142) were included; additionally, all those with positive serology for *Leishmania* (*n* = 187) were included, except in the NUTS3 region of Área Metropolitana do Porto, where a random selection of 15 was made among the positive donors (*n* = 39). A total of 163 participants with positive *Leishmania* serology were included, using these criteria. To complete a total of 800 participants, a random selection was made among those who had a simultaneously negative serology for TOSV and *Leishmania* (numbers were assigned according to their position in the database and drawn using artificial intelligence). This selection considered representativity by NUTS regions—ensuring that at least 20 samples were selected from each NUTS3 region, totaling at least 80 samples per NUTS2 region (Table 1). Inclusion of all/most participants with positive TOSV and/or *Leishmania* serology allowed a more adequate statistical power to assess potential associations between seropositivity for these pathogens and the possible serological cross-reaction particularly between two phleboviruses. Although distinct sand fly species are considered primary vectors for SFSV and TOSV/*Leishmania*, co-exposure in human populations could suggest shared ecological niches, overlapping vector distributions, or common exposure risk factors for these pathogens.

**Table 1:** Distribution of positive results by NUTS 2 and 3 region and estimated adjusted prevalence

Region	Total samples (*n*)	Positive samples (*n*)	Crude prevalence (%)	Adjusted prevalence (%)
Norte	200	11	5.5	8.0
Alto Minho	20	1	5.0	
Cávado	24	0	0.0	
Ave	24	2	8.3	
Área Metropolitana do Porto	42	5	11.9	
Alto Tâmega e Barroso	20	0	0.0	
Tâmega e Sousa	24	1	4.2	
Douro	26	2	7.7	
Terras de Trás-os-Montes	20	0	0.0	
Centro	151	5	3.3	2.9
Região de Aveiro	26	1	3.8	
Região de Coimbra	26	0	0.0	
Região de Leiria	25	0	0.0	
Viseu Dão-Lafões	25	1	4.0	
Beira Baixa	23	0	0.0	
Beiras e Serra da Estrela	26	3	11.5	
Oeste e Vale do Tejo	80	2	2.5	2.7
Oeste	28	1	3.6	
Médio Tejo	26	0	0.0	
Lezíria do Tejo	26	1	3.8	
Grande Lisboa	105	9	8.6	8.6
Península de Setúbal	80	3	3.8	3.8
Alentejo	99	8	8.1	8.3
Alentejo Litoral	21	0	0.0	
Baixo Alentejo	26	3	11.5	
Alto Alentejo	26	2	7.7	
Alentejo Central	26	3	11.5	
Algarve	85	8	9.4	9.4
Total	800	46	5.8	5.7

Categorical variables extracted from the questionnaire were analyzed mostly using the original categories provided as answer options, but regrouping was performed in some cases. Classification of professions was performed using the European Skills, Competences, and Occupations (ESCO) Classification of Occupations, developed by the European Commission since 2010. Classification of parishes as rural or non-rural followed the Portuguese Rural Development Program 2014–2020.

### Serological study

Anti-SFSV antibodies were detected by microneutralization technique. Human sera were heat-inactivated at 56 °C for 30 min, and then diluted from 1:10 to 1:80 and mixed in equal volumes with 1000 50% tissue culture infectious dose (TCID_50_) of SFSV (Sabin strain) in 96-well plates. Following a 1-h incubation at 37 °C, 100 μL of a Vero E6 cell suspension (5 × 10^5^ cells/mL) was added. Each plate included positive and negative controls. After 5 days, cytopathic effect (CPE) was assessed, and neutralization titers were determined. Detection of antibodies in any titer was considered a positive result. A sensitivity of 98.1% and a specificity of 98.8% were assumed for this technique; these were calculated for CPE-based virus neutralization test (VNT) with reference to 90% plaque reduction neutralization test (PRNT_90_) for Zika virus [30] since data for SFSV are not available, but the same technique was used.

Anti-TOSV and anti-*Leishmania* antibodies had previously been detected in these samples, as described in Rocha et al. [28] and [31], respectively.

### Statistical analysis

Crude prevalence was calculated for each NUTS2 and 3 region by dividing the number of positive samples by the total number of samples. To calculate the adjusted prevalence for each NUTS2 region, a correction was applied to the crude prevalence on the basis of the population weight of each NUTS3 region within the corresponding NUTS2 region, considering the population aged 18–65 years. Finally, true prevalence was estimated at national level on the basis of the following formula: true prevalence (TP) = (adjusted prevalence – 1 + specificity)/(sensitivity—1 + specificity).

Absolute and relative frequencies and hypothesis testing were performed using IBM® SPSS® Statistics Version 29.0. Geographical representation and analysis of results was obtained using QGIS® Version 3.22. Descriptive statistics were expressed as absolute frequencies and percentages for categorical variables and as medians with interquartile intervals (IQIs) for asymmetric continuous variables (e.g., age). Missing or unknown data were excluded from denominators, unless stated otherwise. Comparisons between groups were performed using Pearson chi-square test for categorical variables (or Fisher’s exact in case of failure of the assumptions of the χ^2^ test). A value of *p* < 0.05 was considered statistically significant.

Multivariate analysis was conducted to identify sociodemographic factors associated with SFSV infection. This analysis was performed through a multiple binary logistic regression model, analyzing variables with statistical meaning in the univariate analysis (*p* < 0.20) and some biologically relevant or potentially confounding variables. For those variables that remained significant, crude odds ratios (ORs) were updated to adjusted odds ratios (aORs) with 95% CI. The Hosmer–Lemeshow test was used for assessing goodness of fit in each multiple logistic regression model [32].

## Results

### Data and sample collection

In total, 800 participants entered this study, including 142 with positive TOSV serology and 163 with positive *Leishmania* serology. Globally, 206 of the 278 municipalities of mainland Portugal were represented. The median age was 42 years old, and 52.8% of participants were male. Higher education was reported by 35.3% of participants; 72.0% had regular contact with domestic animals, 50.4% owned dogs, and 27.4% mentioned practicing outdoor activities at night. A total of 26.4% reported having nets/screens in some or all the windows or doors at home. Additionally, 50.5% resided in a rural parish, and 23.1% had traveled abroad in the previous 2 years.

### Serological results

**Fig. 1:**
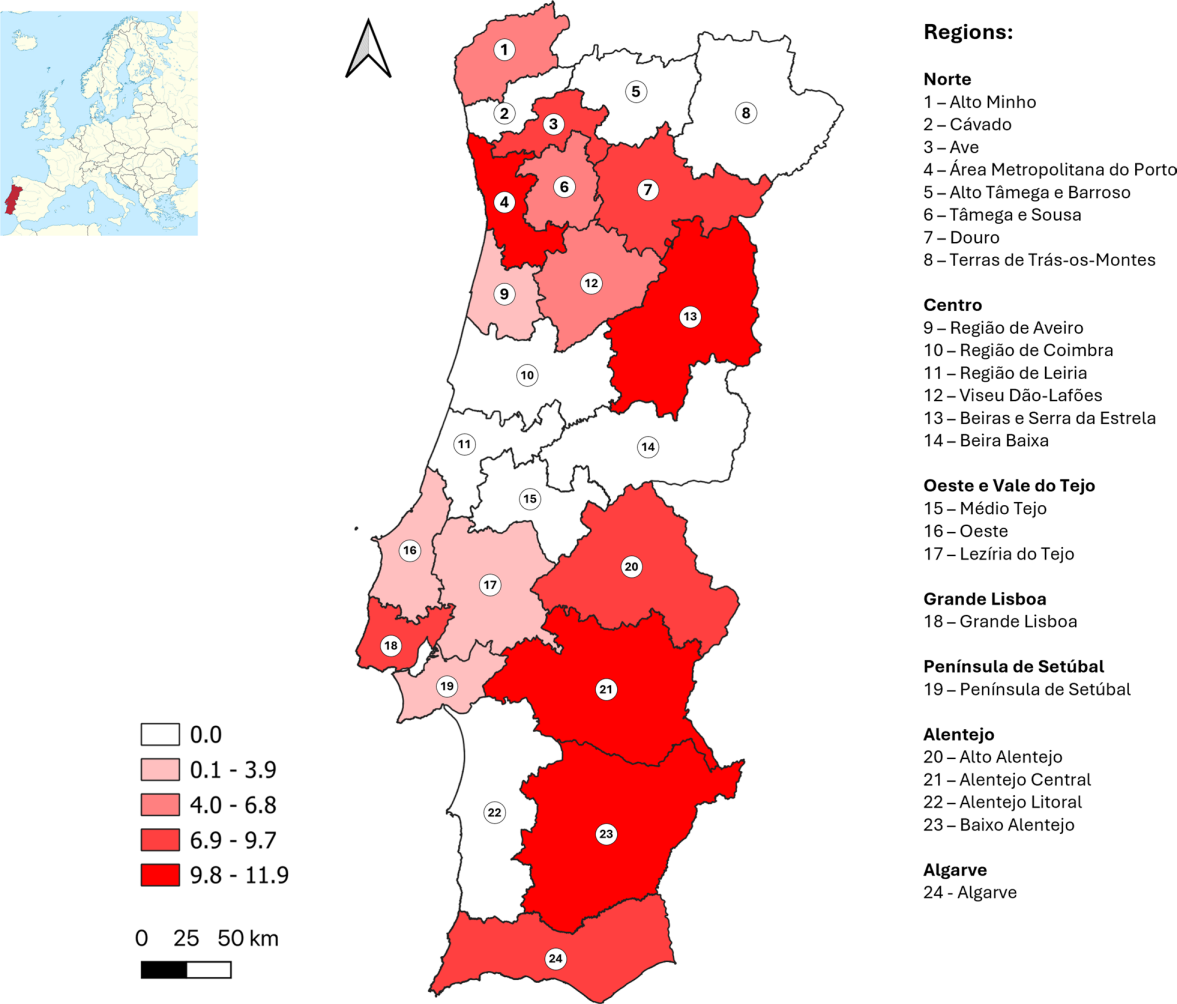
Distribution of estimated SFSV seroprevalence values (%) by NUTS3 region

In total, 46 (5.8%) samples had neutralizing antibodies against SFSV. The distribution of positive results by NUTS2 and 3 regions is represented in Table 1. Figure 1 presents the distribution of crude seroprevalence by NUTS3 region. National estimated true seroprevalence was 4.7% (95% CI 3.4–6.3%). At NUTS3 level, crude seroprevalence values ranged from 0.0% to 11.9%, with the highest seroprevalences in the Área Metropolitana do Porto, Beiras e Serra da Estrela, Alentejo Central, and Baixo Alentejo regions.

**Fig. 2:**
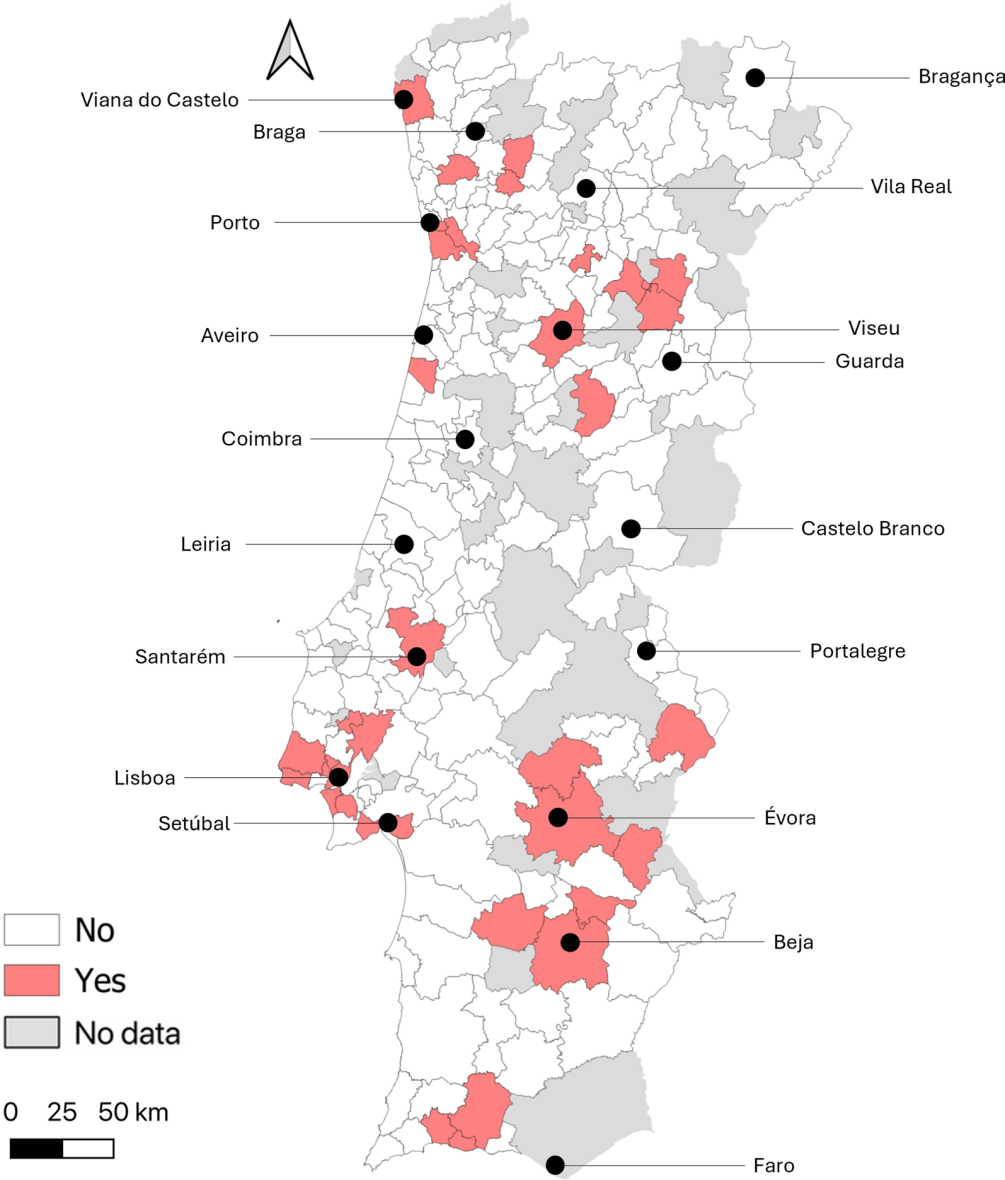
Geographic distribution of blood donors with positive SFSV serology by municipality (the approximate location of district capital cities is highlighted)

Positive blood donors were found in 35 of the 206 municipalities sampled (Fig. 2). Of 16 municipalities with over ten donors tested, the following presented the highest positivity rates, in descending order: Silves (15.8%), Lagoa (15.4%), Vila Nova de Gaia (14.3%), Viana do Castelo (10.0%), Sintra (9.5%), Lisboa (9.4%), and Portimão (9.4%).

### Associations between sociodemographic variables and asymptomatic infection

In univariate analysis, residing in the Algarve, Alentejo, or Grande Lisboa regions and residing in a non-rural parish were significantly associated with a positive serologic result (Table 2). Seropositivity was higher in males and in people older than 49 years old, although these associations did not reach statistical significance. Regular contact with domestic animals, ownership of dogs, positive *Leishmania* serology, or positive TOSV serology were not significantly associated with a positive SFSV serology. In multivariate analysis, the only factor associated with a positive SFSV serologic result was residing in the Algarve, Alentejo, or Grande Lisboa regions (aOR 3.05; 95% CI 1.85–5.02; *p* < 0.001) (Supplementary Table 2).

**Table 2:** Distribution of participants by serological result and by category, for sociodemographic variables

Variables	Categories	Samples, % (*n*)	Seropositive donors, % (*n*/total *N*)	*p*-Value
Sex	Male	52.8 (422/799)	6.9 (29/422)	0.152 (χ^2^ = 2.05; *df * = 1)
	Female	47.2 (377/799)	4.5 (17/377)	
Age (years)	18–24	11.9 (95/798)	4.2 (4/95)	0.845 (χ^2^ = 1.39; *df *= 4)
	25–34	19.9 (159/798)	6.9 (11/159)	
	35–44	27.8 (222/798)	5.0 (11/222)	
	45–54	28.6 (228/798)	6.6 (15/228)	
	55–65	11.8 (94/798)	5.3 (5/94)	
Level of education^a^	1–4	2.5 (20/785)	0.0 (0/20)	0.448 (FET = 3.52)
	5–9	18.2 (143/785)	4.9 (7/143)	
	10–12	43.9 (345/785)	5.2 (18/345)	
	Bachelor’s	26.4 (207/785)	6.8 (14/207)	
	MSc/Ph.D.	8.9 (70/785)	10.0 (7/70)	
Occupation^b^	Student	9.2 (59/640)	5.1 (3/59)	
	Retired	2.0 (13/640)	0.0 (0/13)	
	Unemployed	3.6 (23/640)	4.3 (1/23)	
	0	2.2 (14/640)	7.1 (1/14)	0.895 (FET = 0.62)
	1–3	35.5 (227/640)	6.6 (15/227)	
	4–5	29.1 (186/640)	5.9 (11/186)	
	6–9	18.4 (118/640)	5.1 (6/118)	
Travel abroad (< 2 years previously)	Yes	23.1 (182/787)	5.5 (10/182)	0.882 (χ^2^ = 0.02; ^df^ = 1)
	No	76.9 (605/787)	5.8 (35/605)	
Type of parish of residence	Non-rural	49.5 (396/800)	7.8 (31/396)	0.012* (χ*2* = 6.25; ^df^ = 1)
	Rural	50.5 (404/800)	3.7 (15/404)	
Regular contact with domestic animals	Yes	72.0 (546/758)	5.7 (31/546)	0.810 (χ*2* = 0.06; ^df^ = 1)
	No	28.0 (212/758)	6.1 (13/212)	
Regular contact with wild animals	Yes	4.8 (35/724)	2.9 (1/35)	0.714 (FET = 0.58)
	No	95.2 (689/724)	6.0 (41/689)	
Practice of outdoor activities during nighttime	Yes	27.4 (200/729)	6.0 (12/200)	0.980 (χ*2* = 0.00; ^df^ = 1)
	No	72.6 (529/729)	6.0 (32/529)	
Use of nets/screens in windows/doors	Yes (all/some)	26.4 (200/759)	4.5 (9/200)	0.319 (χ*2* = 0.99; ^df^ = 1)
	None	73.6 (559/759)	6.4 (36/559)	
Ownership of dog(s)	Yes	50.4 (397/787)	5.8 (23/397)	0.950 (χ*2* = 0.00; ^df^ = 1)
	No	49.6 (390/787)	5.9 (23/390)	
Positive *Leishmania* serology	Yes	20.4 (163/800)	5.5 (9/163)	0.888 (χ*2* = 0.02; ^df^ = 1)
	No	79.6 (637/800)	5.8 (37/637)	
Positive TOSV serology	Yes	17.8 (142/800)	7.0 (10/142)	0.466 (χ*2* = 0.53; ^df^ = 1)
	No	82.2 (658/800)	5.5 (36/658)	
NUTS2 region of residence	Norte	25.0 (200/800)	5.5 (11/200)	0.198 (FET = 8.22)
	Centro	18.9 (151/800)	3.3 (5/151)	
	Oeste e Vale do Tejo	10.0 (80/800)	2.5 (2/80)	
	Grande Lisboa	13.1 (105/800)	8.6 (9/105)	
	Península de Setúbal	10.0 (80/800)	3.8 (3/80)	
	Alentejo	12.4 (99/800)	8.1 (8/99)	
	Algarve	10.6 (85/800)	9.4 (8/85)	

^a^Categories refer to the number of years of formal school education completed

^b^Category numbers refer to the category numbers of the European Skills, Competences, and Occupations classifications

^*^Statistically significant

*FET* Fisher’s exact test, *MSc* Master of Science, *NUTS* Nomenclature of Territorial Units for Statistics, *Ph.D.* Doctor of Philosophy, *TOSV* Toscana virus

## Discussion

This study represents the first nationwide SFSV human seroprevalence study in Portugal. National true seroprevalence was estimated at 4.7%, with regional values ranging from 0.0% to 11.9%. A comparison with a previous regional study in which the same serological technique was used shows overlapping positivity rates in the Península de Setúbal (4.3% versus 3.8%; samples collected in 2019) [14] NUTS3 region. Furthermore, the regional seroprevalence estimates from the present study are significantly higher than those reported in other Mediterranean countries, including neighboring Spain, where rates ranged from 0.0% to 5.1% across nine provinces (confirmed by neutralization assays) in a study conducted over two decades ago [6]. However, it should be noted that the present study used a subsample of blood donors, specifically including those who presented positive serology for other sand-fly-borne pathogens, which may have influenced the results. Interestingly, in this sample, the adjusted prevalence was higher for SFSV than for TOSV (5.7% versus 3.7%), which is contrary to findings in humans in other endemic areas where both viruses were simultaneously tested [7], but is concordant with a previous study in dogs in Portugal [18].

Factors explaining SFSV seroprevalence in Portugal could be related to aspects of the presence, abundance, and infection rate of the vectors of SFSV in the country. *P. papatasi* has only been detected in the Algarve region in recent years. *P. ariasi* has been identified in several of the prospected municipalities in northern and central Portugal, including in Porto, and this could explain the higher SFSV prevalence found in Área Metropolitana do Porto and Ave regions, where higher humidity and lower summer temperatures could be more favorable for *P. ariasi*. *Phlebotomus perniciosus* seems to be widespread in Portugal [26], according to data from national surveillance (2016–2023), and this species could also play a role in SFSV transmission. However, SFSV has, to date, not been detected in sand flies collected in Portugal.

In this study, a likely intense exposure to SFSV is shown in areas where it had not previously been documented, namely in the Alentejo, Algarve, and Grande Lisboa NUTS2 regions. Although some of these areas overlap with regions with higher *Leishmania* and/or TOSV seroprevalence, no statistically significant association was found between a positive individual serology for these pathogens and SFSV. This could indicate that the vectors of SFSV in Portugal could effectively differ from *Leishmania* and TOSV vectors, although the ecological niches for all of them could be similar. One study showed, however, a serological association between *L. infantum* and SFSV in sheltered dogs from southern Portugal [17]. Different studies showed conflicting results regarding the association between SFSV and TOSV seropositivity [10, 12]. Additional studies could provide further insights into the vector species and the environmental and sociodemographic factors associated with the differential exposure to these sand-fly-borne pathogens in Portugal. Recent studies have increasingly recognized the presence of other phleboviruses, such as Massilia and Alcube viruses, in certain regions of Portugal, particularly in the Península de Setúbal and Algarve areas, highlighting the complex phleboviral landscape in these endemic regions [33].

In this study, no factors were found to be significantly associated with a seropositive SFSV result besides geographic location of residence. In previous studies, increasing age [34] and female sex [14] have been associated, although inconsistently, with a positive serology for SFSV. Additionally, travel history may play a role in seropositivity, as exposure can occur outside the place of residence [35]. In the present study, no significant association was found between a positive result and travel abroad in the previous 2 years; however, travel to different regions within the country was not assessed. Residing in a non-rural area was associated with positive serology in univariate analysis, which could be partially explained by the endophilic behavior of *P. papatasi* [36], the vector of SFSV. This species can thrive in human-inhabited environments in Europe [37], potentially increasing exposure risk in non-rural areas. However, this association did not remain significant in multivariate analysis, likely due to confounding with regional factors such as the high seroprevalence observed in the Grande Lisboa region, where most parishes are classified as non-rural.

In summary, these findings and the previous studies, suggest that, in Portugal, raised clinical awareness for SFSV should be promoted, especially in the Algarve, Alentejo, and Grande Lisboa regions. SFSV should be considered in the differential diagnosis of undifferentiated febrile syndrome, especially in the months of May–October, considering knowledge on the seasonal activity of phlebotomine sand flies in Portugal [38]. This clinical approach will also rely on the availability of molecular biology and serology techniques to detect SFSV in endemic areas. Neutralization-based assays are generally regarded as the gold standard for the confirmation of *Phlebovirus* antibody specificity [39], but these techniques are labor-intensive and require live viruses, limiting their wide-scale implementation. Alongside raising clinical awareness in these regions, it is equally important to enhance public education on personal protective measures, such as the use of repellents, as well as environmental management strategies of living spaces to reduce suitable vector breeding sites.

In spite of the many caveats for the use of serology for individual determination of previous SFSV infection status, the detection of antibodies can be relevant from a public health perspective, especially when comparing the findings between different regions and by crossing the results with distribution of human cases and evidence from SFSV in mammals and vectors, following a One Health approach. This knowledge could be helpful in controlling human SFSV infections, highlighting regions where environmental interventions to reduce phlebotomine breeding sites and increased adherence to the use of arthropod repellent by humans (from dusk to dawn) could be recommended. Future research should focus on understanding the specific ecologic niches of SFSV in Portugal.

Assessment of sand-fly-borne phleboviruses in blood donors could be relevant beyond epidemiological surveillance. The detection of antibodies in blood donors raises concerns about the potential transmission of sand-fly-borne phleboviruses to virus-naive individuals through blood transfusions or organ transplantation. Although, to the authors’ knowledge, there are no confirmed cases of transfusion- or transplant-associated transmission of SFSV or other phleboviruses such as TOSV, the possibility remains a theoretical risk. This highlights the importance of continued surveillance and further research to assess potential implications for blood and organ safety, particularly in endemic areas.

These findings cannot be transposed to the general Portuguese mainland population, since only healthy people aged 18–65 years were included, and the profile of people who donate blood could be different from the age-matched general population in each region and between regions. In addition, the representativeness even of the blood donor population itself could have been affected by the difficulty in obtaining a truly probabilistic sample, due to logistic constraints in some regions. The timing of sample collection should also be considered when interpreting these results. As most samples were collected before the typical sand fly activity season in Portugal (May–October), the detected antibodies likely reflect exposure from previous seasons. However, 13.8% of the samples were collected during May or June, which could coincide with the early phase of sand fly activity. While this may allow for the possibility of recent infections in a small number of cases, overall seropositivity in this study is presumed to represent past exposures.

## Conclusions

In conclusion, this study provides the first national estimate of SFSV seroprevalence in mainland Portugal, revealing a true prevalence of 4.7%, with significant regional variation. These findings suggest a wider circulation of SFSV in the country than previously recognized, particularly in the Algarve, Alentejo, and Grande Lisboa regions. Despite no clear associations with sociodemographic factors beyond geographic area of residence, the study underscores the need for increased clinical awareness of SFSV, especially in cases of febrile illness during peak months for sand fly activity. The absence of a significant association with TOSV or *Leishmania* seropositivity reinforces that distinct vector species may contribute to SFSV transmission in Portugal, warranting further entomological studies. Future research should focus on clarifying the specific vectors involved, improving diagnostic capabilities, and assessing the clinical impact of SFSV infections in Portugal.

## Supplementary Information

Additional file 1. Summary of the methodology of the previous study—cross-sectional study on the prevalence of anti-*Leishmania* antibodies in blood donors in mainland Portugal [28].

Additional file 2. Potential risk factors for SFSV infection, according to logistic regression models to estimate crude and adjusted odds ratio values.

## Data Availability

The datasets generated and analyzed during the current study are not publicly available due to confidentiality commitment with the participants, as stated in the consent declaration, but are available from the corresponding author on reasonable request.

## References

[1] Simmonds P, Adriaenssens EM, Lefkowitz EJ, Oksanen HM, Siddell SG, Zerbini FM, et al. Changes to virus taxonomy and the ICTV statutes ratified by the international committee on taxonomy of viruses (2024). Arch Virol. 2024;169:236. 39488803 10.1007/s00705-024-06143-y PMC11532311

[2] Lambert AJ, Hughes HR. Clinically important phleboviruses and their detection in human samples. Viruses. 2021;13:1500. 34452365 10.3390/v13081500 PMC8402687

[3] Calisher CH, Calzolari M. Taxonomy of phleboviruses, emphasizing those that are sandfly-borne. Viruses. 2021;13:918. 34063467 10.3390/v13050918 PMC8156068

[4] Maroli M, Feliciangeli MD, Bichaud L, Charrel RN, Gradoni L. Phlebotomine sandflies and the spreading of leishmaniases and other diseases of public health concern. Med Vet Entomol. 2013;27:123–47. 22924419 10.1111/j.1365-2915.2012.01034.x

[5] Konstantinou GN, Papa A, Antoniadis A. Sandfly-fever outbreak in Cyprus: are phleboviruses still a health problem? Travel Med Infect Dis. 2007;5:239–42. 17574146 10.1016/j.tmaid.2007.02.002

[6] Mendoza-Montero J, Gámez-Rueda MI, Navarro-Marí JM, De La Rosa-Fraile M, Oyonarte-Gómez S. Infections due to sandfly fever virus serotype Toscana in Spain. Clin Inf Dis. 1998;27:434–6. 10.1086/514684 9770137

[7] Ortuño M, Muñoz C, Spitzová T, Sumova P, Iborra MA, Pérez-Cutillas P, et al. Exposure to *Phlebotomus perniciosus* sandfly vectors is positively associated with Toscana virus and *Leishmania infantum* infection in human blood donors in Murcia Region, southeast Spain. Transbound Emerg Dis. 2022;69:e1854–64. 35357094 10.1111/tbed.14520 PMC9790518

[8] Bichaud L, Piarroux RP, Izri A, Ninove L, Mary C, de Lamballerie X, et al. Low seroprevalence of sandfly fever Sicilian virus antibodies in humans Marseille France. Clin Microbiol Inf. 2011;17:1189–90. 10.1111/j.1469-0691.2011.03509.x 21595791

[9] Masse S, Ayhan N, Capai L, Bosseur F, de Lamballerie X, Charrel R, et al. Circulation of Toscana virus in a sample population of Corsica, France. Viruses. 2019;11:817. 31487870 10.3390/v11090817 PMC6784206

[10] Calamusa G, Valenti RM, Vitale F, Mammina C, Romano N, Goedert JJ, et al. Seroprevalence of and risk factors for Toscana and Sicilian virus infection in a sample population of Sicily (Italy). J Inf. 2012;64:212–7. 10.1016/j.jinf.2011.11.012 PMC3630500 22120113

[11] Cusi MG, Gandolfo C, Valentini M, Savellini GG. Seroprevalence of antibodies to sandfly fever Sicilian virus in a sample population in Tuscany Italy. Vector-Borne Zoo Dis. 2013;13:345. 10.1089/vbz.2011.0945 23289397

[12] Polat C, Ayhan N, Saygan MB, Karahan S, Charrel R, Ergünay K. Comprehensive cross-sectional evaluation of human sandfly-borne *Phlebovirus* exposure in an endemic region. Viruses. 2023;15:1902. 37766308 10.3390/v15091902 PMC10535931

[13] Ayari R, Chaouch H, Findlay-Wilson S, Hachfi W, Ben Lasfar N, Bellazreg F, et al. Seroprevalence and risk factors associated with phleboviruses and Crimean-Congo hemorrhagic fever virus among blood donors in central Tunisia. Pathogens. 2024;13:348. 38668303 10.3390/pathogens13040348 PMC11054088

[14] Maia C, Ayhan N, Cristóvão JM, Pereira A, Charrel R. Human seroprevalence of Toscana virus and Sicilian phlebovirus in the southwest of Portugal. Eur J Clin Microbiol Infect Dis. 2022;41:137–41. 34389911 10.1007/s10096-021-04332-0

[15] Bento Guerra A, Gouveia C, Zé-Zé L, Amaro F, Cordeiro Ferreira G, Brito MJ. Prolonged febrile illness caused by Sicilian virus infection in Portugal. Sweden: Malmo; 2018.

[16] Pereira A, Ayhan N, Cristóvão JM, Vilhena H, Martins Â, Cachola P, et al. Antibody response to Toscana virus and sandfly fever Sicilian virus in cats naturally exposed to phlebotomine sand fly bites in Portugal. Microorganisms. 2019;7:339. 31514266 10.3390/microorganisms7090339 PMC6780191

[17] Maia C, Alwassouf S, Cristóvão JM, Ayhan N, Pereira A, Charrel RN, et al. Serological association between *Leishmania infantum* and sand fly fever Sicilian (but not Toscana) virus in sheltered dogs from southern Portugal. Parasit Vectors. 2017;10:92. 28285587 10.1186/s13071-017-2023-x PMC5346850

[18] Alwassouf S, Maia C, Ayhan N, Coimbra M, Cristovao JM, Richet H, et al. Neutralization-based seroprevalence of Toscana virus and sandfly fever Sicilian virus in dogs and cats from Portugal. J Gen Virol. 2016;97:2816–23. 27589865 10.1099/jgv.0.000592

[19] Xhekaj B, Kurum E, Stefanovska J, Cvetkovikj A, Sherifi K, Rexhepi A, et al. Neutralization-based seroprevalence of Toscana virus and sandfly fever Sicilian virus in dogs in the Republic of Kosovo. Parasit Vectors. 2025;18:48. 39930491 10.1186/s13071-025-06681-7 PMC11812177

[20] Alwassouf S, Christodoulou V, Bichaud L, Ntais P, Mazeris A, Antoniou M, et al. Seroprevalence of sandfly-borne phleboviruses belonging to three serocomplexes (sandfly fever Naples, sandfly fever Sicilian and Salehabad) in dogs from Greece and Cyprus using neutralization test. PLoS Negl Trop Dis. 2016;10:e0005063. 27783676 10.1371/journal.pntd.0005063 PMC5081206

[21] Ayhan N, Sherifi K, Taraku A, Bërxholi K, Charrel RN. High rates of neutralizing antibodies to Toscana and sandfly fever Sicilian viruses in livestock. Kosovo Emerg Infect Dis. 2017;23:989–92. 28518045 10.3201/eid2306.161929 PMC5443445

[22] Horton KC, Wasfy M, Samaha H, Abdel-Rahman B, Safwat S, Abdel Fadeel M, et al. Serosurvey for zoonotic viral and bacterial pathogens among slaughtered livestock in Egypt. Vector-Borne and Zoonotic Diseases. 2014;14:633–9. 25198525 10.1089/vbz.2013.1525 PMC4676263

[23] Ayhan N, Rodríguez-Teijeiro JD, López-Roig M, Vinyoles D, Ferreres JA, Monastiri A, et al. High rates of antibodies against Toscana and Sicilian phleboviruses in common quail *Coturnix coturnix* birds. Front Microbiol. 2022;13:1091908. 36687574 10.3389/fmicb.2022.1091908 PMC9846092

[24] Muñoz C, Ayhan N, Ortuño M, Ortiz J, Gould EA, Maia C, et al. Experimental infection of dogs with Toscana virus and sandfly fever Sicilian virus to determine their potential as possible vertebrate hosts. Microorganisms. 2020;8:596. 32326097 10.3390/microorganisms8040596 PMC7232252

[25] Ayhan N, Charrel NR. Sandfly-borne viruses of demonstrated/relevant medical importance. London: IntechOpen; 2019.

[26] Centro de Estudos de Vetores e Doenças Infeciosas Doutor Francisco Cambournac. Relatório REVIVE 2023—Culicídeos, Ixodídeos e Flebótomos: Rede de Vigilância de Vetores. 2024.

[27] de Freitas MT, Maia C. *Phlebotomus perniciosus*. Trends Parasitol. 2024;40:649–50. 38704297 10.1016/j.pt.2024.04.007

[28] Rocha R, Gonçalves L, Conceição C, Andrade P, Cristóvão JM, Condeço J, et al. Prevalence of asymptomatic *Leishmania* infection and knowledge, perceptions, and practices in blood donors in mainland Portugal. Parasit Vectors. 2023;16:357. 37817278 10.1186/s13071-023-05980-1 PMC10563231

[29] Instituto Nacional de Estatística (INE). Resultados Provisórios. 2021. Resultados Provisórios—Censos 2021.

[30] Nurtop E, Villarroel PMS, Pastorino B, Ninove L, Drexler JF, Roca Y, et al. Combination of ELISA screening and seroneutralisation tests to expedite Zika virus seroprevalence studies. Virol J. 2018;15:192. 30587193 10.1186/s12985-018-1105-5 PMC6307276

[31] Rocha R, Kurum E, Ayhan N, Charrel R, Maia C. Seroprevalence of Toscana virus in blood donors in mainland Portugal. Parasit Vectors. 2025;18:82. 40033431 10.1186/s13071-025-06726-x PMC11874794

[32] Fagerland MW, Hosmer DW. A generalized Hosmer-Lemeshow goodness-of-fit test for multinomial logistic regression models. Stata J. 2012;12:447.

[33] Amaro F, Zé-Zé L, Alves MJ. Sandfly-Borne phleboviruses in Portugal: four and still counting. viruses. 2022;14:1768. 36016390 10.3390/v14081768 PMC9413822

[34] Al-numaani SA, Al-Nemari AT, El-Kafrawy SA, Hassan AM, Tolah AM, Alghanmi M, et al. Seroprevalence of Toscana and sandfly fever Sicilian viruses in humans and livestock animals from western Saudi Arabia. One Health. 2023;17:100601. 37520847 10.1016/j.onehlt.2023.100601 PMC10372353

[35] Becker M, Zielen S, Schwarz T, Linde R, Hofmann D. Pappataci-Fieber. Klin Padiatr. 1997;209:377–9. 9445923 10.1055/s-2008-1043979

[36] Orshan L, Szekely D, Khalfa Z, Bitton S. Distribution and seasonality of *Phlebotomus* sand flies in cutaneous leishmaniasis foci, Judean Desert. Israel J Med Entomol. 2010;47:319–28. 20496578 10.1603/me09096

[37] Dantas-Torres F, Latrofa M, Otranto D. Occurrence and genetic variability of *Phlebotomus papatasi* in an urban area of southern Italy. Parasit Vectors. 2010;3:77. 20738865 10.1186/1756-3305-3-77 PMC2936393

[38] Zé-Zé L, Amaro F, Osório HC, Giovanetti M, Lourenço J, Alves MJ. Molecular identification and ecology of Portuguese wild-caught phlebotomine sandfly specimens. Zoo Dis. 2022;2:19–31.

[39] Ergünay K, Litzba N, Lo MM, Aydoğan S, Saygan MB, Us D, et al. Performance of various commercial assays for the detection of Toscana virus antibodies. Vector-Borne Zoonotic Dis. 2011;11:781–7. 21395410 10.1089/vbz.2010.0224 PMC3115411

