# Supplementary material for: Seroprevalence of sand fly fever Sicilian virus in blood donors in mainland Portugal
Source: Parasit Vectors. 2025 Jul 5;18:261. doi: 10.1186/s13071-025-06885-x (PMC12228258; doi:10.1186/s13071-025-06885-x)
Supplement: Supplementary file 2 — Additional file 2. Potential risk factors for SFSV infection, according to logistic regression models to estimate crude and adjusted odds ratio values. [file 13071_2025_6885_MOESM2_ESM.docx]

| Potential Risk Factor | | Univariate | | | | | | | Multivariate | | | | | |  |  |  |
| --- | --- | --- | --- | --- | --- | --- | --- | --- | --- | --- | --- | --- | --- | --- | --- | --- | --- |
|  |  | % in Sample | | Crude OR | | | 95% CI | | Adjusted OR | | 95% CI | | *p*-value | |  |  |  |
| Age ≥ 49 years old | | 25.8 | | 1.75 | | | 0.94-3.25 | | 1.76 | | 0.92-3.36 | | 0.088 | |  |  |  |
| Male sex | | 52.8 | | 1.56 | | | 0.84-2.89 | | 1.46 | | 0.77-2.76 | | 0.245 | |  |  |  |
| Residing in GL, Algarve or Alentejo region | | 36.1 | | 2.21 | | | 1.21-4.02 | | 1.82 | | 1.02-3.40 | | **0.048*** | |  |  |  |
| Residing in a non-rural parish | | 50.5 | | 2.20 | | | 1.17-4.15 | | 1.75 | | 0.90-3.40 | | 0.098 | |  |  |  |
| Higher education level | | 35.3 | | 1.58 | | | 0.87-2.89 | | 1.68 | | 0.88-3.19 | | 0.116 | |  |  |  |
| Constant | |  | |  | | |  | | 0.049 | |  | | <0.001 | |  |  |  |
| Hosmer and Lemeshow Test | |  | | |  |  | | | | Sig.=0.947 | | | | | |  |  |
|  | |  | |  | | |  | |  | | | |  | |  | | |
| Abbreviations: CI - confidence interval; GL - Grande Lisboa; OR - odds ratio. | | | |  | | |  | |  | | | |  | |  | | |
| *Statistically significant | |  | |  | | |  | |  | | | |  | |  | | |

**Supplementary table 2.** Potential risk factors for SFSV infection, according to logistic regression models to estimate crude and adjusted odds ratio values.
